# Gender bias in autism screening: measurement invariance of different model frameworks of the Autism Spectrum Quotient

**DOI:** 10.1192/bjo.2023.562

**Published:** 2023-10-02

**Authors:** Hannah L. Belcher, Nora Uglik-Marucha, Silia Vitoratou, Ruth M. Ford, Sharon Morein-Zamir

**Affiliations:** Institute of Psychiatry, Psychology and Neuroscience, King's College London, UK; Psychometrics and Measurement Laboratory, Department of Biostatistics and Health Informatics, Institute of Psychiatry, Psychology and Neuroscience, King's College London, UK; School of Psychology and Sports Science, Anglia Ruskin University, UK

**Keywords:** Autistic spectrum disorders, neurodevelopmental disorders, psychological testing, statistical methodology, community mental health teams

## Abstract

**Background:**

The Autism Spectrum Quotient is a popular autism screening tool recommended for identifying potential cases of autism. However, many women with autism demonstrate a different presentation of traits to those currently captured by screening measures and assessment methods, such as the Autism Spectrum Quotient.

**Aims:**

Different models of the Autism Spectrum Quotient have been proposed in the literature, utilising different items from the original 50-item scale. Within good-fitting models, the current study aimed to explore whether these items assess autistic traits similarly across men and women.

**Method:**

Seventeen Autism Spectrum Quotient models were identified from the literature. Using the responses of a large sample of adults from the UK general population (5246 women, 1830 men), confirmatory factor analysis was used to evaluate the fit of each model. Measurement invariance with respect to gender, adjusting for age, was explored in the 11 model frameworks that were found to have satisfactory fit to our data.

**Results:**

It emerged that only two items were gender invariant (non-biased), whereas for the remaining items, the probability of endorsement was influenced by gender. In particular, women had a higher probability of endorsing items relating to social skills and communication.

**Conclusions:**

If the items of the Autism Spectrum Quotient indeed reflect autism-related traits, those items should be rephrased to ensure they do not present a gender-related bias. This is vital for ensuring more timely diagnoses and support for all people with autism.

Despite growing public awareness of autism in females, girls and women continue to be diagnosed significantly later in life on average than boys and men.^1–4^ This may be partly because of a different presentation of autistic traits in women and girls, including fewer social communication difficulties,^5–7^ and less restrictive and repetitive behaviours and interests.^8,9^ Although there is evidence that males may be more likely to develop autism because of sex-specific gene mutations and epigenetic changes, as well as the involvement of sex chromosomes and hormone involvement,^10^ females may be more likely to go undiagnosed because they experience greater social pressure to ‘fit in’ and hide their autistic traits.^11,12^ Moreover, clinicians may be biased against identifying autism in females,^13^ and females may need to present with additional difficulties to meet diagnostic criteria.^14^

There are approximately three males to every one female diagnosed with autism,^15^ but the gender disparity in the actual incidence of autism is predicted to be lower when undiagnosed cases are considered.^16^ Missed or late autism diagnoses are of concern, given the heightened risk of co-occurring mental health conditions and suicidal behaviours experienced by people with autism.^17,18^ Yet many adults endure multiple referrals to different health professionals before receiving a diagnosis of autism spectrum condition (ASC),^19^ and this occurs more often in women.^1^ Earlier diagnosis and support can have a considerable beneficial effect on quality of life in adulthood and facilitate better psychiatric management of co-occurring mental health difficulties in later life.^20,21^

Screening measures, such as the Autism Spectrum Quotient, play a significant role in identifying individuals for autism diagnosis,^22^ and the short ten-item version is recommended by the National Institute for Health and Care Excellence for the screening and assessment of adults.^23^ Several different model frameworks have been proposed in the literature for the Autism Spectrum Quotient items. The most popular are the original 50-item, five-factor version (social skills, attention switching, attention to detail, communication and imagination)^22^ and the AQ-10, which includes ten items from the original scale and has been assumed to measure a unitary construct of autism,^24^ although some studies suggest more than one factor.^25^ However, a previous examination of the predictive value of the Autism Spectrum Quotient on actual autism diagnoses found that 64% of those scoring below the cut-off were ‘false negatives’, meaning that a high percentage of people with autism may be at risk of not receiving a referral for a diagnostic assessment.^26^

There is growing concern in the literature that screening tools such as the Autism Spectrum Quotient may operate differently based on the gender of the respondent, and this might explain the historical overrepresentation of men and boys in autism research.^27^ That is, screening tools can be gender biased, and that would lead to non-valid measurements and prevalence estimations, underestimating autism in women and girls. In psychometrics, this is called measurement bias, or non-invariance (non-equivalence) or differential item functioning. If a measure is to be used to screen all individuals, then it should provide the same measurement for the same amount of the latent trait (autism) regardless of one's group membership (gender). Otherwise, measurement non-invariance of the tool can lead to different clinical decisions based solely on gender,^28^ and hinder meaningful group comparisons as it can overestimate or underestimate symptoms in one group compared with another.^29,30^ In such cases, it becomes necessary to consider implementing group-specific cut-off points, removing biased items from the instrument or even discontinuing the use of the tool altogether.^28^

To date, limited research has been conducted into gender-related measurement invariance of different models of the Autism Spectrum Quotient. For instance, the AQ-10^24^ underwent two such investigations using item response theory.^31,32^ An initial study identified one item biased against women and another biased against men, concluding that both genders were subject to bias at the item level, which cancelled out any overall test score bias.^31^ However, a subsequent replication found only one item biased against women, which was not observed in the initial study, and no bias was observed at the test score level.^32^ To our knowledge, no such investigations have been conducted in the original 50-item model. Additional and more comprehensive investigations of gender-related measurement invariance are necessary to ensure that Autism Spectrum Quotient models, especially those utilised in screening and clinical decision-making, promote fair referral for diagnostic assessments and the provision of appropriate support, regardless of one's gender. Notably, no previous study has examined if there is gender bias in the items under all published model frameworks while simultaneously considering the influence of age.

## Aims

The current study explores whether the measures of autistic traits, as conceptualised through different Autism Spectrum Quotient models, assess the autistic traits similarly for men and women, using a large sample of individuals from the general population. The study is novel in that it tests the different theoretical models available in the literature (17 in total), both separately by gender and combined. It also investigates measurement invariance with respect to gender, and adjusts for age for models whose theoretical structure was confirmed in our data (nine models) or whose use in the literature is prevalent (two models). Taking into account the age of the participants is particularly important because of the different developmental trajectories experienced by women and men with autism.^33^ Thus, this study represents the most comprehensive evaluation of gender-related measurement invariance in the Autism Spectrum Quotient to date. It distinguishes itself from previous research by considering the influence of age, and benefits from a significantly larger sample size compared with most prior studies investigating various Autism Spectrum Quotient models, enhancing the robustness of its findings.

## Method

### Participants

Our sample was derived from a previous study in which we collected data from the general population by administering the Autism Spectrum Quotient. The current study used data from all participants regardless of Autism Spectrum Quotient scores and diagnosis. Participants were adults in the UK who were invited to contribute to the nationwide study examining gender differences in social behaviours and mental health (see Results for participant demographics and characteristics).^1^

### Measure

#### Autism Spectrum Quotient

The Autism Spectrum Quotient was originally developed as a 50-item scale to screen individuals for possible ASC.^22^ Each of the items is scored on a four-point scale from ‘definitely agree’ to ‘definitely disagree’, and maps onto five domains (communication, social skills, imagination, attention to detail and attention switching). Cronbach's alpha coefficients of each of these dimensions is moderate to high (0.63–0.77). The degree of association between two time points of the scale's administration is reported to be strong (*r* = 0.7). A cut-off score of ≥32 has been found to be accurate in identifying possible cases of ASC in the general population.^22^

#### Other models of the Autism Spectrum Quotient

The first model of the Autism Spectrum Quotient that was proposed as an alternative to the original model by Baron-Cohen et al^22^ was a three-factor model with 26 items.^34^ Following Austin, further models using either all items or a subset of items derived from the original scale have been developed and validated. In the literature, we identified 17 such models, with varying numbers of items, factors and model complexity (see Supplementary Appendix 1 available at https://doi.org/10.1192/bjo.2023.562 for mapped items and their corresponding factors). The number of items used across the models ranges from 6 to 50, and the factors vary from one to five, with four models also utilising hierarchical^35,36^ or bifactor solutions.^37^ Just over half of the items did not consistently load on the same factors across models. For instance, item AQ10 (‘In social groups, I can easily keep track of several people's conversations’) loaded three times on a ‘repetitive behaviours and routines/attention switching’ factor, two times on the ‘social skills’ factor, and five times on the ‘communication’ factor in the ten models where it was used.

### Procedure

The current study used participants’ scores on the Autism Spectrum Quotient. Participants completed the survey online, using Qualtrics. At the start of the survey, they were presented with the information sheet followed by consent to participate statements, and were requested to indicate that they agreed to before progressing to the questionnaires. The questionnaires allowed one response per person and items could not be skipped. All procedures involving human patients were approved by the corresponding author's institute (Anglia Ruskin University; approval number FST/FREP/13/402).

### Transparency and openness

Raw and coded data and analysis code is available upon request to the corresponding author. No part of this study was pre-registered before submission.

### Statistical analyses

This study evaluated measurement invariance of the Autism Spectrum Quotient items with respect to gender, adjusting for age, subject to models that have been proposed in the literature to date. First, all 17 models identified from the literature were fitted to assess their goodness-of-fit indices. The 11 models that had close fit in our data (see below) were subsequently used to test measurement invariance. In two instances where models did not have close fit, the measurement invariance was analysed because of the wide use of those models in the literature based on the number of citations in Google Scholar (>100).

#### Dimensionality

Confirmatory factor analysis (CFA) for categorical data was used to study the fit of each model, both separately by gender (configural models), and when the data were combined. The weighted least squares mean and variance adjusted^38^ estimator was used. A broad set of goodness-of-fit indices were used to evaluate model fit to account for model complexity, including the root mean square error of approximation (RMSEA),^39^ comparative fit index (CFI),^40^ Tucker–Lewis index (TLI)^41^ and standardised root mean residual.^39^ RMSEA values <0.05 indicate close fit, and values <0.08 suggest adequate fit. The CFI and TLI range from 0 to 1, with values >0.95 indicating close fit.

#### Measurement invariance

**Fig. 1:**
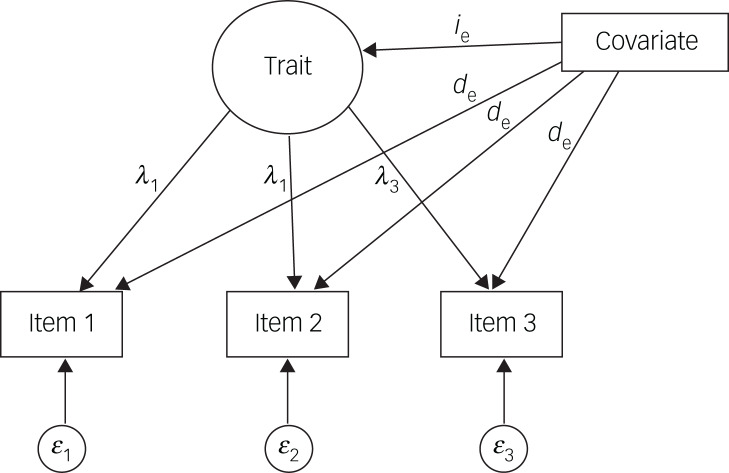
Multiple indicators, multiple causes model path diagram. Rectangles denote observed variables, such as the items (e.g. the Autism Spectrum Quotient items) and the exogenous covariates (e.g. age or gender). Circles represent latent variables, such as latent trait(s) (e.g. autism spectrum condition) and item-specific measurement error (*ε*). Arrows linking the latent variable with items denote item factor loadings (*λ*_s_), and arrows connecting the covariate with items (*d*_e_) and the latent variable (*i*_e_) signify direct and indirect effects, respectively.

The multiple indicators, multiple causes model (MIMIC)^42,43^ is a CFA model with exogenous covariates. For measurement invariance testing, the items and the latent factors are regressed onto one or more exogenous covariate(s), which can be either numerical (such as age in years) or categorical (such as gender) (see Fig. 1 for MIMIC model path diagram). The direct effects of the exogenous covariate on the items are estimated. A significant direct effect on an observed item signifies measurement non-invariance. Measurement non-invariance points to differential item functioning for selected groups or covariate levels. That is, for the same levels of the latent trait, group membership alone affects the probability of endorsing a particular item, thus introducing group bias in the measurement. For instance, for the same levels of a trait, an item can result in different probability of endorsement of the item with respect to the participant's gender. Similar interpretation holds in the case of a continuous exogenous covariate (such as age), where the values of the covariate alter the probability of endorsement.

In this study, the MIMIC was used to evaluate measurement invariance on 11 models, which were selected either because they had close fit to the data or because of their popularity in the literature.^22,24^ Each model assumes a different number of latent traits, some consisting of different items, thus reflecting different traits. That being the case, measurement invariance refers to adjusting the probability of endorsement adjusting for different trait(s), allowing for a plethora of investigations, under a different setting for each item. All analyses were conducted in MPlus (version 8.5 for Mac OS X).^44^

## Results

### Demographic characteristics

A total of 7179 individuals from the UK general population participated in this study. With respect to gender, 5246 (74.1%) individuals identified as women and 1830 (26.9%) identified as men. Participants who identified as transgender (*n* = 55) or other (*n* = 48) were removed as the numbers were too small for the current analysis. There were no missing responses.

The analyses were conducted on 7076 participants. Mean age was 32.22 years (s.d. = 13.8), with 71.5% of participants aged <40 years. There were 380 (5.3%; 4.2% women) participants who reported a diagnosis of ASC. The most reported psychiatric diagnoses from the whole sample were depression (25.2%), general anxiety disorder (12%), social anxiety disorder (5.1%), intellectual disability (4.2%) and eating disorder (4%).

### CFA

CFA was used to evaluate the fit of 17 models of the Autism Spectrum Quotient on the complete data, as well as separately by gender.

The corresponding goodness-of-fit indices are presented in Table 1. Nine models had close fit, with the best being Jia et al,^46^ which assumes two factors (‘Social communication’ and ‘attention to detail’) and uses nine items. One further model had adequate fit,^24^ and five models had less than adequate fit in the data. Finally, two models by Bertrams^52^ showed empirical under-identification because of the inclusion of less than the minimum number of items per factor required for identification.

**Table 1:** Confirmatory factor analysis goodness-of-fit indices for each competing model

Model	Items	Factors	Entire sample (*N* = 7076)		Women (*n* = 5246)	Men (*n* = 1830)
Close fit						
Austin (2005)^34^	26	3 first order: ‘social skills’, ‘details/patterns’ and ‘communication/mindreading'	RMSEA	0.087	0.088	0.081
			CFI	0.920	0.922	0.924
			TLI	0.912	0.915	0.916
			SRMR	0.062	0.061	0.065
Freeth et al (2013)^45^	35	4 first order: ‘social situation enjoyment’, ‘social communication’, ‘attention to detail’ and ‘imagination'	RMSEA	0.077	0.078	0.074
			CFI	0.905	0.907	0.902
			TLI	0.898	0.900	0.894
			SRMR	0.063	0.063	0.069
Hoekstra et al (2011)^36^	28	1 second order: ‘social behaviour’; 4 first order: ‘social skills’, ‘routine’, ‘switching’, ‘imagination’; separate first order: ‘numbers and patterns’	RMSEA	0.076	0.079	0.071
			CFI	0.909	0.909	0.906
			TLI	0.900	0.900	0.897
			SRMR	0.058	0.060	0.061
Jia et al (2019)^46^	9	2 first order: ‘social communication’ and ‘attention to detail'	RMSEA	0.045	0.046	0.045
			CFI	0.994	0.994	0.992
			TLI	0.991	0.991	0.990
			SRMR	0.023	0.023	0.027
Kloosterman et al (2011)^47^	28	5 first order: ‘social skills’, ‘communication/mindreading’, ‘restricted/repetitive behaviour’, ‘imagination’ and ‘attention to detail’	RMSEA	0.073	0.073	0.073
			CFI	0.933	0.936	0.927
			TLI	0.926	0.928	0.919
			SRMR	0.054	0.054	0.061
Lau et al (2013)^48^	35	5 first order: ‘socialness’, ‘mind- reading’, ‘patterns’, ‘attention to detail’ and ‘attention switching’	RMSEA	0.077	0.076	0.077
			CFI	0.903	0.909	0.889
			TLI	0.895	0.902	0.880
			SRMR	0.061	0.059	0.070
Lau et al (2013)^49^	39	5 first order: ‘sociability’, ‘social cognition’, ‘narrow focus’, ‘interest in patterns’ and ‘resistance to change’	RMSEA	0.069	0.069	0.069
			CFI	0.908	0.914	0.897
			TLI	0.902	0.908	0.890
			SRMR	0.055	0.054	0.063
Murray et al (2017)^37^	28	[Bifactor] A general factor (excluding ‘number/patterns’ items) and 5 specific factors: ‘social skills’, ‘routine’, ‘switching’, ‘imagination’ and ‘numbers/patterns’	RMSEA	0.066	0.066	0.067
			CFI	0.936	0.941	0.923
			TLI	0.925	0.930	0.91
			SRMR	0.048	0.047	0.053
Russel-Smith et al (2011)^50^	28	3 first order: ‘social skills’, ‘communication/mindreading’, ‘details/patterns’	RMSEA	0.069	0.069	0.067
			CFI	0.948	0.949	0.944
			TLI	0.943	0.944	0.939
			SRMR	0.051	0.051	0.057
Adequate fit						
Allison et al (2012)^24^	10	1 first order: ‘unitary construct of autism’	RMSEA	0.093	0.099	0.088
			CFI	0.942	0.939	0.937
			TLI	0.926	0.922	0.919
			SRMR	0.043	0.044	0.044
Less than adequate fit						
Baron-Cohen et al (2001)^22^	50	5 first order: ‘social skills’, ‘attention switching’, ‘attention to detail’, ‘communication’ and ‘imagination’	RMSEA	0.086	0.086	0.082
			CFI	0.792	0.802	0.783
			TLI	0.781	0.791	0.772
			SRMR	0.079	0.078	0.086
Hoekstra et al (2008)^35^	50	1 second order: ‘social interaction’; 4 first order: ‘social skills’, ‘communication’, ‘attention switching’ and ‘imagination’; 1 separate first order: ‘attention to detail’	RMSEA	0.086	0.086	0.082
			CFI	0.791	0.801	0.784
			TLI	0.782	0.791	0.774
			SRMR	0.079	0.079	0.086
Murray et al (2017)^37^	50	[Bifactor] A general factor (excluding ‘attention to detail’ items) and 5 specific factors: ‘social skills’, ‘attention switching’, ‘attention to detail’, ‘communication’ and ‘imagination’	RMSEA	0.072	0.072	0.069
			CFI	0.859	0.868	0.853
			TLI	0.846	0.857	0.84
			SRMR	0.063	0.062	0.069
Russel-Smith et al (2011)^50^	38	4 first order: ‘social skills’, ‘details/patterns’, ‘understanding others/communication’ and ‘imagination’	RMSEA	0.076	0.077	0.070
			CFI	0.894	0.894	0.898
			TLI	0.886	0.887	0.891
			SRMR	0.066	0.067	0.069
Stewart and Austin (2009)^51^	43	4 first order: ‘socialness’, ‘pattern’, ‘understanding others/communication’ and ‘imagination’	RMSEA	0.080	0.081	0.074
			CFI	0.857	0.859	0.862
			TLI	0.849	0.851	0.855
			SRMR	0.071	0.072	0.075

First-order factors are derived from the covariation among the observed variables. Second-order factors account for the covariation among the multiple first-order factors. RMSEA, root mean square error of approximation; CFI, comparative fit index; TLI, Tucker–Lewis index; SRMR, standardised root mean residual.

### Measurement invariance

The nine models with close fit to the data as indicated by CFA underwent measurement invariance analysis with respect to gender, adjusted for age (Table 2). Two additional models by Allison et al^24^ and Baron-Cohen et al^22^ were also included because of their popularity in research and clinical practice.

**Table 2:** DSM-5 criteria for each item and frequency of measurement non-invariant items present in models used in relation to gender adjusted for age and model latent trait(s) levels

Item	Label	DSM-5 criteria	Number of models used	Number of non-invariant entries	More likely to be endorsed by	Percentage of non-invariance (%)
AQ01	I prefer to do things with others than on my own	A1	8	1	Women	12.50
AQ02	I prefer to do things the same way over and over again^a^	B2	5	2	Either (1:1 women:men)	40
AQ03	If I try to imagine something, I find it very easy to create a picture in my mind	None	5	3	Men	60
AQ04	I frequently get so strongly absorbed in one thing that I lose sight of other things^a^	B3	6	4	Men	66.67
AQ05	I often notice small sounds when others do not^a^	B4	8	7	Women	88
AQ06	I usually notice car number plates or similar strings of information^a^	B3	10	7	Either (2:5 men:women)	70
AQ07	Other people frequently tell me that what I've said is impolite, even though I think it is polite^a^	A3	5	1	Men	20
AQ08	When I'm reading a story, I can easily imagine what the characters might look like	None	6	5	Women	83
AQ09	I am fascinated by dates^a^	B3	8	6	Either	75
AQ10	In social group, I can easily keep track of several people's conversations	A1	8	4	Women	50
AQ11	I find social situations easy	None	9	1	Men	11.11
AQ12	I tend to notice details that other do not^a^	B3	8	2	Men	25
AQ13	I would rather go to a library than a party^a^	None	8	8	Women	100
AQ14	I find making up stories easy	None	4	4	Men	100
AQ15	I find myself drawn more strongly to people than to things	A3	9	9	Women	100
AQ16	I tend to have very strong interests which I get upset about if I can't pursue^a^	B3	4	3	Men	75
AQ17	I enjoy social chit-chat	None	8	7	Women	87.50
AQ18	When I talk, it isn't always easy for others to get a word in edgeways^a^	A1	2	2	Women	100
AQ19	I am fascinated by numbers^a^	B3	10	10	Men	100
AQ20	When I'm reading a story, I find it difficult to work out the character's intentions^a^	None	10	9	Either (1:8 women:men)	90
AQ21	I don't particularly enjoy reading fiction^a^	None	2	2	Men	100
AQ22	I find it hard to make new friends^a^	None	10	1	Women	10
AQ23	I notice patterns in things all the time^a^	B3	10	4	Men	40
AQ24	I would rather go to the theatre than a museum	None	1	1	Women	100
AQ25	It does not upset me if my daily routine is disturbed	B2	6	4	Men	66.67
AQ26	I frequently find that I don't know how to keep a conversation going^a^	A1	6	5	Men	83.33
AQ27	I find it easy to ‘read between the lines’ when someone is talking to me	None	7	0	Neither	0
AQ28	I usually concentrate more on the whole picture, rather than the small details	B3	4	4	Men	100
AQ29	I am not very good at remembering phone numbers	B3	2	2	Men	100
AQ30	I don't usually notice small changes in a situation, or a person's appearance	B2	2	2	Men	100
AQ31	I know how to tell if someone listening to me is getting bored	A1	6	0	Neither	0
AQ32	I find it easy to do more than one thing at once	None	7	7	Women	100
AQ33	When I talk on the phone, I'm not sure when it's my turn to speak^a^	A1	3	3	Women	100
AQ34	I enjoy doing things spontaneously	B2	8	3	Either (1:2 women:men)	37
AQ35	I am often the last to understand the point of a joke^a^	A3	4	4	Women	100
AQ36	I find it easy to work out what someone is thinking or feeling just by looking at their face	A2	9	9	Women	100
AQ37	If there is an interruption, I can switch back to what I was doing very quickly	B2	7	5	Either (1:4 women:men)	71.43
AQ38	I am good at social chit-chat	A1	7	6	Women	86
AQ39	People often tell me that I keep going on and on about the same thing^a^	A1	6	3	Women	50
AQ40	When I was young, I used to enjoy playing games involving pretending with other children	A3	3	2	Women	67
AQ41	I like to collect information about categories of things^a^	B3	8	8	Men	100
AQ42	I find it difficult to imagine what it would be like to be someone else^a^	None	5	2	Either (1:1 women:men)	40
AQ43	I like to plan any activities I participate in carefully^a^	B2	3	1	Women	33
AQ44	I enjoy social occasions	None	10	1	Men	10
AQ45	I find it difficult to work out people's intentions^a^	None	10	7	Women	70
AQ46	New situations make me anxious^a^	None	7	6	Women	85.71
AQ47	I enjoy meeting new people	None	10	1	Men	10
AQ48	I am a good diplomat	None	5	2	Men	40
AQ49	I am not very good at remembering people's date of birth	B3	1	1	Men	100
AQ50	I find it very easy to play games with children that involve pretending	A3	6	4	Women	66.67

a. Item is reverse coded.

We tested whether the probability of endorsing an item is influenced by gender (women versus men), adjusted for age. A positive significant direct effect indicates that an item is less likely to be endorsed by women compared with men (reference group), as the rating scale of the items ranges from 0 (definitely agree) to 3 (definitely disagree). For an item that is reverse coded (denoted by *), a positive significant direct effect indicates that the item is more likely to be endorsed by women. Significant direct effects of gender, adjusted for age, for each model are shown in Supplementary Appendix 2.

Table 2 presents all 50 items that were assessed in this study. Most items are included in multiple models. For instance, item AQ19 (‘I am fascinated by numbers’) was present in ten models out of the 11 evaluated. Table 2 also presents the number of models for which an item was found to be non-invariant (i.e. biased with respect to gender, adjusted for the model trait(s) and age). Using the previous example, item AQ19 was non-invariant in all ten models in which it appeared (i.e. 100% non-invariant). In contrast, the probability of endorsement of item A22 (‘I find it hard to make new friends’) was found to be biased with respect to gender in only one model out of the ten in which it was included (10%).

**Fig. 2:**
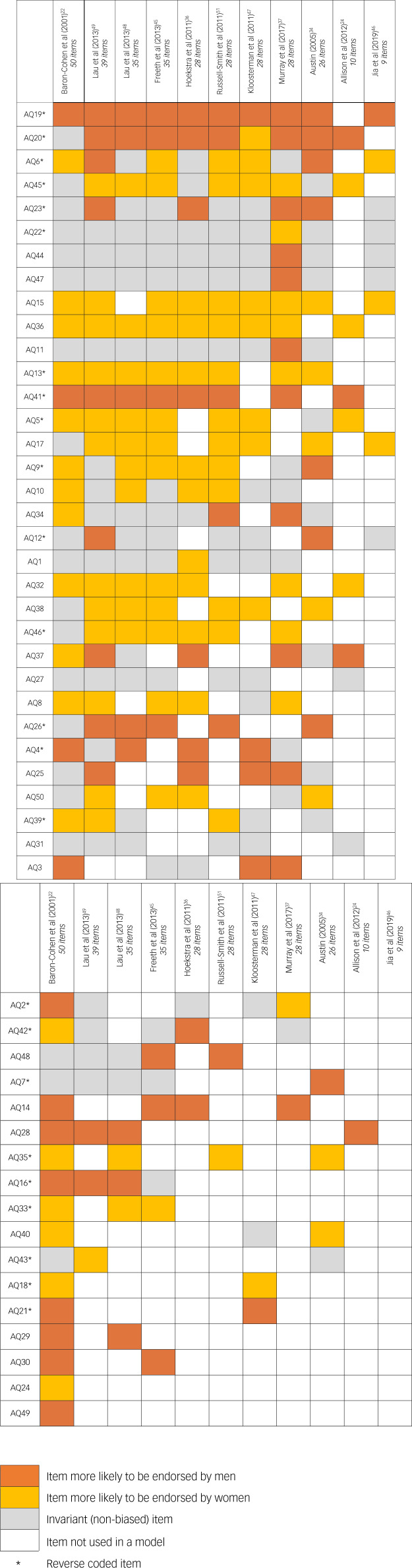
A graphical visualisation of frequency of measurement non-invariant (biased) items across all models, sorted by decreasing number of models an item was used in and the number of non-invariant entries. Orange squares denote the frequency each item was more likely to be endorsed by men (e.g. item AQ19 was more likely to be endorsed by men in all ten models it featured in), yellow squares signify the frequency an item was more likely to be endorsed by women (e.g. item AQ45 was more likely to be endorsed by women in seven out of the ten models it featured in), grey squares represent the frequency an item was invariant (non-biased; e.g. item AQ45 was used in ten models, across which it was invariant three times) and white squares denote that an item was not used in a particular model.

Only two items, AQ27 (‘I find it easy to ‘read between the lines’ when someone is talking to me’) and AQ31 (‘I know how to tell if someone listening to me is getting bored’), were consistently invariant with respect to gender adjusted for age (see also Fig. 2).

Table 2 also presents each item against the DSM-5 core criteria (a: social communication/interaction domain; b: restricted and repetitive behaviour domain), and its subdomains for ASC. The number of items mapping onto each subdomain varied. Eighteen of the items did not map onto any of the core criteria or subdomains. Women were more likely to endorse items on the social communication/interaction domain, whereas men were more likely to endorse items on the restricted and repetitive behaviour domain.

## Discussion

The aim of the current study was to explore whether items on the Autism Spectrum Quotient assess autistics traits similarly across men and women, using a large sample of individuals from the general population, across 11 model frameworks. To our knowledge, this is the first study to look at gender invariance on the Autism Spectrum Quotient, adjusted for age, across multiple models presented in the literature. Our findings indicate that 20 items were consistently biased toward men (13 in at least 50% of the models present) and 21 items were consistently biased toward women (18 in at least 50% of the models present). Examining the wording of the traits assessed among the items that were more likely to be endorsed by women, ten were indicative of autistic traits (e.g. item AQ5: ‘I often notice small sounds others do not’), and 11 items indicated typically non-autistic traits (e.g. AQ1: ‘I prefer to do things with others than on my own’). In comparison, nine of the items more likely to be endorsed by men were indicative of autistic traits (e.g. AQ16: ‘I tend to have very strong interests which I get upset about if I can't pursue’), whereas 11 indicated typically non-autistic traits (e.g. AQ11: ‘I find social situations easy’).

Using the factor model for the original 50-item Autism Spectrum Quotient,^22^ analysis revealed that when items were biased in at least 50% of the models, women were more likely to endorse all four gender-biased items of the ‘social skills’ factor and six of the seven items of the ‘communication’ factor. In contrast, men were more likely to endorse five out of eight items of the ‘attention to detail’ factor, four of the nine items from the ‘imagination’ factor and three of the seven items from the ‘attention switching’ factor.

The biased (non-invariant) items that are more likely to be endorsed by women compared with men reflect a gender difference in the presentation of autistic traits, which screening measures like the AQ-10 are not sensitive to. For example, women were more likely than men with the same traits (as measured by the Autism Spectrum Quotient) and age to say that (a) they preferred doing things with others rather than alone, (b) they could keep track of multiple conversations in social situations, (c) they were drawn more strongly to people rather than things, (d) they enjoyed social conversation and felt that they were good at it, (e) they could tell what someone was thinking or feeling from their facial expressions and (f) they enjoyed playing pretend games with others as children. This indicates a greater propensity for women to engage in social activities and social communication than men. These traits represent an opposing profile to that suspected in autism cases, where it is assumed that there is a consistent deficit in these areas. Our analysis did not examine differences between autistic and non-autistic groups, but it can be hypothesised from previous literature that these areas of socio-communication also may remain relatively intact for women with autism compared with men.^1^ Women with autism demonstrate fewer socio-communication difficulties than men on common ASC assessment measures despite demonstrating similar levels of childhood autistic traits.^9,53^ Additionally, girls with autism have been found to be more likely than boys to engage in reciprocal conversation and imaginative play typical for their developmental age.^5^ This may be because of differences in how girls and boys are socialised and gender stereotypes that are often reinforced by care givers, encouraging females to be more sociable and empathic.^54–56^ A particular concern is that developmental screening measures are initially developed and tested on clinical populations, which often present a male gender bias in themselves.^57^ For example, the Autism Spectrum Quotient was originally developed with a clinical sample of 48 men with autism and only 13 women with autism.^22^ Consequently, different presentations of autistic traits, as seen in women and those diagnosed late, were not apparent to the researchers.^11^

Our findings align, to some extent, with previous studies investigating gender-related measurement invariance in different models of the Autism Spectrum Quotient. The notable deviations between our results and those reported in the literature may be attributed to the adjustment for age in our analysis. Specifically, in relation to the AQ-10 model, Murray et al^31^ identified gender bias in two items (AQ28 and AQ32), whereas a subsequent study by Murray et al^32^ found bias in only one item (AQ41), which was not observed in the initial study. In contrast, our analysis involved a more comprehensive examination of the items across multiple models as well as adjusting for age, leading us to identify additional gender non-invariant items within the AQ-10. Based on our data, a total of eight items (AQ5, AQ20, AQ28, AQ32, AQ36, AQ37, AQ41, AQ45) exhibited gender bias. In a separate examination of gender-related measurement invariance within the AQ-28 model among the autistic population,^36^ Grove et al^58^ identified gender bias in only two items (AQ13 and AQ46). In our investigation, we observed a much larger number of biased items, specifically 19 (see Fig. 2). However, it is worth noting that Grove et al^58^ utilised a sample of men and women with autism, raising the question of whether the substantially fewer biased items found in their study is attributable to the diminished effect of gender on items within the autistic population.

Another investigation into a newer and less frequently used model of the Autism Spectrum Quotient, the AQ-9 model,^46^ employed multiple-group CFA to evaluate the scale for gender bias. The findings supported the configural invariance (equivalence of the basic structure of the measurement model) and metric invariance (the strength of the relationship between the items and the underlying construct, as indicated by factor loadings, is equivalent across genders) of the model. However, scalar invariance was not established, suggesting that women and men had different expected responses to the items for the same absolute trait levels. These results align with the findings of our study, which revealed four items (AQ6, AQ15, AQ17, AQ19) with different expected responses for women and men when controlling for the same levels of autistic traits. However, Jia et al^46^ did not investigate which items contributed to scalar non-invariance, and considered the measure to be free from gender bias based on the support for metric invariance. It is worth noting that all three levels of measurement invariance (configural, metric and scalar) are prerequisites for meaningful group comparisons based on observed scores.^30^ Our study employed multiple models across a much larger sample of the general public, making it the most comprehensive and robust examination of item invariance of the Autism Spectrum Quotient to date. We argue that these items demonstrate that the different models of the Autism Spectrum Quotient do not assess autistic traits equally for both genders, and that gender alone can bias scores on the scale. This bias calls into question the conceptual construct of the measure. Given that different items will be more likely endorsed by some but not others and considering the uneven distribution of gender-related item bias across different dimensions of autistic traits, it indicates that the measure may no longer be regarded as reliably assessing the full diagnostic criteria of autism across men and women as intended. This may explain the reported low predictive value of the measure.^26^

Furthermore, the items on the full Autism Spectrum Quotient do not fully represent either the DSM-5 core traits (broad construct) or subdomains (narrow construct).^59^ Although gender bias on individual Autism Spectrum Quotient items (behaviour exemplars) may be irrelevant if the scale as a whole is able to measure narrow and broad constructs equally across genders, our results do not support this. Importantly, there is an unequal number of items across the narrow constructs; for example, the subdomain A2 (‘Nonverbal communication problems, such as abnormal eye contact, posture, facial expressions, tone of voice and gestures, and an inability to understand these’) and B4 (‘Hyper-or hyperreactivity to sensory input or unusual interest in sensory aspects of the environment’) only have one item each. However, there are 18 items that do not map onto the DSM-5 criteria at all, such as item 13 (‘I would rather go to a library than a party’) and item 21 (‘I don't particularly enjoy reading fiction)’. Looking at the broader construct, women were more likely to endorse items on the ‘social communication/interaction domain’ (both positively and negatively) and men were more likely to endorse items on the ‘restricted and repetitive behaviour domain’ (both positively and negatively). This suggests a bias in the behaviour exemplars being used to capture the narrow and broader constructs. Previous research stresses the importance of using item analyses to determine whether gender biases exist at the narrow construct level, and if so, there is a need for developing new instruments with gender-dependent criteria.^27^ This would involve collecting a wider range of behavioural exemplars beyond classic autistic traits, and exploring co-existing features (e.g. masking), that better represent the narrow and broader autism construct for all genders.

Our findings stress the importance of revising the Autism Spectrum Quotient to ensure that it measures the full range of autistic traits in all individuals. This is vital to ensuring that individuals with autism will not miss appropriate assessments because of their initial scores on the Autism Spectrum Quotient at screening. We also warn against the use of the Autism Spectrum Quotient in research settings comparing men and women, given that it is unable to measure identical sets of traits in both. As the current study examined all 50 items across multiple different models, other researchers may benefit from utilising these findings in revising existing autism measures or developing new ones. Furthermore, future research should examine measurement invariance in the autistic population, as it is not known whether the effect of gender on the items of the Autism Spectrum Quotient under different models either diminishes or may even be exacerbated in the autistic population.

### Limitations

The current work does not investigate all levels of measurement invariance with respect to gender. The MIMIC model used in the analysis – because of the need to control for age – can only test the invariance of item thresholds (often referred to as scaler or strong invariance). More thorough investigations are required to assess all levels of measurement invariance with respect to gender, using multiple-group CFA. Additionally, although gender-related item bias of the Autism Spectrum Quotient is evident, additional examination may be necessary to determine the extent to which the presence of biased items affects the screening performance itself. Nevertheless, the presence of item bias raises concerns about the validity of the construct measured by the Autism Spectrum Quotient, as our findings suggest that it assesses different traits for women and men. Furthermore, the gender-related item bias is not evenly distributed across factors, indicating that the measure should not be regarded as assessing the full diagnostic criteria of autism across men and women.

### Constraints on generality

First, this analysis was conducted on UK participants and ethnicity was not recorded, meaning that these findings may not be generalisable to people with autism living in other countries or of different ethnicities. Although there is consensus among different nationalities on the diagnostic criteria of ASC, research suggests that there may be cultural differences in what is considered neurotypical development.^60,61^ Indeed, one historic problem is that research on autism assessment and screening does not account for cultural differences. Social communication differences are considered a core trait of the condition; however, cultural factors can also contribute to social communication differences.^62^ It is also important to acknowledge the inequalities that exist in gaining ASC diagnoses for racial and ethnic minority groups. There is a disparity in diagnosis between high-income countries and low- and middle-income countries resulting from a lack of culturally appropriate screening and diagnostic tools,^63^ and there is evidence that children from racial and ethnic minority groups are more likely to be misdiagnosed or diagnosed later compared with White children, because of increased stigma, lack of education and lack of screening and interventions within those communities.^64^ Future research exploring the screening of ASC should actively recruit and record a racially and ethnically diverse population to measure cultural invariance, and further analysis should be conducted on different translations of the Autism Spectrum Quotient in different countries.

Second, the study was only able to compare those identifying as men or women, and although we allowed participants to choose their own gender identity rather than being reduced to their biological gender, there were too few individuals identifying as transgender or ‘other’ to be included within the analysis. However, a large number of people with autism identify as gender diverse.^65^ Item invariance of the Autism Spectrum Quotient should also be investigated in these populations.

## Supporting information

Belcher et al. supplementary material Belcher et al. supplementary material

## Data Availability

The data that support the findings of this study are available from the corresponding author, H.L.B., upon reasonable request.
