# Supplementary material for: Gender bias in autism screening: measurement invariance of different model frameworks of the Autism Spectrum Quotient
Source: BJPsych Open. 2023 Oct 2;9(5):e173. doi: 10.1192/bjo.2023.562 (PMC10594186; doi:10.1192/bjo.2023.562)
Supplement: Belcher et al. supplementary material [file S2056472423005628sup001.docx]

**Appendix 1.** A summary of items and factors they belong to for each of the 17 models investigated.

| **Factors** | |
| --- | --- |
| S | Social factors |
| C | Communication factors |
| A | Attention, patterns & details factor |
| I | Imagination factor |
| R | Repetitive behaviours & routines/Attention switching |
| ASD | General ASD factor |
|  | Item not assigned to a factor |

| **Model** | **Baron-Cohen et al. (2001)** | **Austin (2005)** | **Hoekstra et al. (2008)** | **Stewart & Austin (2009)** | **Russell-Smith et al. (2011a)** | **Russell-Smith et al. (2011b)** | **Hoekstra et al. (2011)** | **Kloosterman et al. (2011)** | **Allison et al. (2012)** | **Lau et al. (2013a)** | **Lau et al. (2013b)** | **Freeth et al. (2013)** | **Murray et al. (2015a)** | **Murray et al. (2015b)** | **Jia et al. (2019)** | **Bertrams (2021a)** | **Bertrams (2021b)** |
| --- | --- | --- | --- | --- | --- | --- | --- | --- | --- | --- | --- | --- | --- | --- | --- | --- | --- |
| **Items** | 50 | 26 | 50 | 43 | 28 | 38 | 28 | 28 | 10 | 35 | 39 | 35 | 50 | 28 | 9 | 6 | 6 |
| **Factors** | 5 | 3 | 2^a^ | 4 | 3 | 4 | 2^b^ | 5 | 5 | 5 | 5 | 4 | Bifactor^b^ | Bifactor^b^ | 2 | 1^c^ | 1^c^ |
| AQ1 | S |  | S | S | S | S | S | S |  | S | S | S | ASD | ASD |  |  |  |
| AQ2* | R |  | R | C |  |  | R^1^ | R |  |  | R |  | ASD | ASD |  |  |  |
| AQ3 | I |  | I | I |  | I | I | I |  |  |  | I | ASD | ASD |  |  |  |
| AQ4* | R |  | R | I |  | I | R^2^ | R |  | R | A^1^ |  | ASD | ASD |  |  |  |
| AQ5* | A | A | A | A | A | A |  | A | A | A^2^ | A^1^ | A | A |  |  |  |  |
| AQ6* | A | A | A | A | A | A | A | A |  | A1 | A^2^ | A | A | A | A |  |  |
| AQ7* | C | C | C | C |  |  |  |  |  | C | A^1^ | C | ASD |  |  |  |  |
| AQ8 | I |  | I | I |  | I | I | I |  |  | C | I | ASD | ASD |  |  |  |
| AQ9* | A | A | A | A | A | A | A |  |  | A^1^ | A^2^ | A | A | A |  |  |  |
| AQ10 | R |  | R | C | S | C | R^2^ | C |  | S | C | C | ASD | ASD |  |  |  |
| AQ11 | S | S | S | S | S | S | S | S |  | S | S | S | ASD | ASD |  |  |  |
| AQ12* | A | A | A | A | A | A |  | A |  | A^2^ | A^1^ | A | A |  | A |  |  |
| AQ13* | S | S | S | S | S | S | S |  |  | S | S | S | ASD | ASD |  |  |  |
| AQ14 | I |  | I | I |  | I | I |  |  |  |  | I | ASD | ASD |  |  |  |
| AQ15 | S | S | S | S | S | S | S | S |  |  | S | S | ASD | ASD | S&C |  |  |
| AQ16* | R |  | R |  |  | A |  |  |  | R | A^1^ | A | ASD |  |  |  |  |
| AQ17 | C | S | C | S | S | S |  | S |  | S | S | S | ASD |  | S&C |  |  |
| AQ18* | C |  | C | S |  |  |  | R |  |  |  |  | ASD |  |  |  |  |
| AQ19* | A | A | A | A | A | A | A | A |  | A^1^ | A^2^ | A | A | A | A |  |  |
| AQ20* | I | C | I | C | C | C | I | I | I | C | C | C | ASD | ASD |  |  |  |
| AQ21* | I |  | I | C |  | I |  | I |  |  |  |  | ASD |  |  |  |  |
| AQ22* | S | S | S | S | S | S | S | S |  | S | S | S | ASD | ASD | S&C |  |  |
| AQ23* | A | A | A | A | A | A | A | A |  | A^2^ | A^1^ | A | A | A | A |  |  |
| AQ24 | I |  | I |  |  |  |  |  |  |  |  |  | ASD |  |  |  |  |
| AQ25 | R | A | R |  |  |  | R^1^ | R |  |  | R |  | ASD | ASD |  |  |  |
| AQ26* | C | S | C | S | S | S |  |  |  | S | S | S | ASD |  |  |  |  |
| AQ27 | C |  | C | C | C | C |  | C | C | C | C | C | ASD |  |  | C | C&S |
| AQ28 | A |  | A |  |  |  |  |  | A | A^2^ | C |  | A |  |  |  |  |
| AQ29 | A |  | A | A |  | A |  |  |  | A^1^ |  |  | A |  |  |  |  |
| AQ30 | A |  | A | C |  | C |  |  |  |  |  | C | A |  |  |  |  |
| AQ31 | C |  | C | C | C | C |  | C | C | C | C |  | ASD |  |  | C | C&S |
| AQ32 | R |  | R | C |  | C | R^2^ |  | R | R | C | C | ASD | ASD |  | R | R |
| AQ33* | C |  | C | C |  |  |  |  |  | C |  | C | ASD |  |  |  |  |
| AQ34 | R | S | R |  | S | S | R^1^ |  |  | R | R | I | ASD | ASD |  |  |  |
| AQ35* | C | C | C | C | C | C |  |  |  | C |  |  | ASD |  |  |  |  |
| AQ36 | S |  | S | C | C | C | I | C | S | C | C | C | ASD | ASD |  | S | C&S |
| AQ37 | R | C | R | C |  | C | R^2^ |  | R | R | C |  | ASD | ASD |  | R | R |
| AQ38 | C | S | C | S | S | S |  | S |  | S | S | S | ASD |  |  |  |  |
| AQ39* | C | C | C | C | C |  |  | R |  | R | A^1^ |  | A |  |  |  |  |
| AQ40 | I | S | I | I |  | I |  | I |  |  |  |  | ASD |  |  |  |  |
| AQ41* | I |  | I | A | A | A | A |  | I | A^1^ | A^2^ | A | ASD | A |  |  |  |
| AQ42* | I |  | I |  |  |  | I |  |  |  | C | C | ASD | ASD |  |  |  |
| AQ43* | R | A | R |  |  |  |  |  |  |  | R |  | ASD |  |  |  |  |
| AQ44 | S | S | S | S | S | S | S | S |  | S | S | S | ASD | ASD | S&C |  |  |
| AQ45* | S | C | S | C | C | C | I | C | S | C | C | C | ASD | ASD |  | S | C&S |
| AQ46* | R |  | R | S | S | S | S/R^1^ |  |  | S | S | S | ASD | ASD |  |  |  |
| AQ47 | S | S | S | S | S | S | S | S |  | S | S | S | ASD | ASD | S&C |  |  |
| AQ48 | S |  | S | C | C |  |  |  |  | S | S | C | ASD |  |  |  |  |
| AQ49 | A |  | A | I |  |  |  |  |  |  |  |  | ASD |  |  |  |  |
| AQ50 | I | S | I | I |  | I | I |  |  |  | S | I | ASD | ASD |  |  |  |

^*^ Item is reverse coded; ^a^ A second-order factor consisting of four first-order factors; ^b^ A general factor and 5 specific factors; ^c^ A second-order factor consisting of three first-order factors; R^1^ denotes ‘routine’ part of factor ‘R’ that was split into two; R^2^ represents ‘attention switching’ part of factor ‘R’ that was split into two; A^1^ denotes ‘pattern’ (Lau et al., 2013a) and ‘narrow focus’ (Lau et al., 2013b) part of factor ‘A’ that was split into two; A^2^ represents ‘attention to detail’ (Lau et al., 2013a) and ‘interest in patterns’ (Lau et al., 2013b) part of factor ‘A” that was split into two.

**Appendix 2.** Significant direct effects of gender adjusted for age for 11 models of AQ (N=7076) – MIMIC model.

| **Model** | **Baron-Cohen et al. (2001)** | **Austin (2005)** | **Hoekstra et al. (2011)** | **Russell-Smith et al. (2011a)** | **Kloosterman et al. (2011)** | **Allison et al. (2012)** | **Lau et al. (2013a)** | **Lau et al. (2013b)** | **Freeth et al. (2013)** | **Murray et al. (2015b)** | **Jia et al. (2019)** |
| --- | --- | --- | --- | --- | --- | --- | --- | --- | --- | --- | --- |
| AQ1 |  |  | -0.055 |  |  |  |  |  |  |  |  |
| AQ2* | -0.076 |  |  |  |  |  |  |  |  | 0.199 |  |
| AQ3 | 0.153 |  |  |  | 0.217 |  |  |  |  | 0.159 |  |
| AQ4* | -0.23 |  | -0.101 |  | -0.14 |  | -0.146 |  |  |  |  |
| AQ5* | 0.278 |  |  | 0.272 | 0.275 | 0.158 | 0.278 | 0.174 | 0.26 |  |  |
| AQ6* | 0.081 | -0.238 |  | 0.079 | 0.074 |  |  | -0.073 | 0.063 |  | 0.07 |
| AQ7* |  | -0.175 |  |  |  |  |  |  |  |  |  |
| AQ8 | -0.081 |  | -0.197 |  |  |  |  | -0.125 | -0.23 | -0.201 |  |
| AQ9* | 0.139 | -0.147 | 0.07 | 0.14 |  |  | 0.067 |  | 0.126 |  |  |
| AQ10 | -0.256 |  | -0.126 | -0.091 |  |  | -0.096 |  |  |  |  |
| AQ11 |  |  |  |  |  |  |  |  |  | 0.323 |  |
| AQ12* |  | -0.272 |  |  |  |  |  | -0.088 |  |  |  |
| AQ13* | 0.139 | 0.129 | 0.12 | 0.139 |  |  | 0.14 | 0.14 | 0.14 | 0.31 |  |
| AQ14 | 0.228 |  | 0.151 |  |  |  |  |  | 0.128 | 0.236 |  |
| AQ15 | -0.176 | -0.182 | -0.194 | -0.173 | -0.175 |  |  | -0.174 | -0.174 | -0.106 | -0.173 |
| AQ16* | -0.295 |  |  |  |  |  | -0.216 | -0.072 |  |  |  |
| AQ17 |  | -0.122 |  | -0.108 | -0.11 |  | -0.108 | -0.107 | -0.108 |  | -0.106 |
| AQ18* | 0.22 |  |  |  | 0.167 |  |  |  |  |  |  |
| AQ19* | -0.294 | -0.595 | -0.367 | -0.293 | -0.309 |  | -0.345 | -0.441 | -0.308 | -0.606 | -0.309 |
| AQ20* |  | -0.201 | -0.17 | -0.085 | 0.079 | -0.085 | -0.075 | -0.079 | -0.069 | -0.106 |  |
| AQ21* | -0.252 |  |  |  | -0.213 |  |  |  |  |  |  |
| AQ22* |  |  |  |  |  |  |  |  |  | 0.153 |  |
| AQ23* |  | -0.379 | -0.1 |  |  |  |  | -0.14 |  | -0.229 |  |
| AQ24 | -0.267 |  |  |  |  |  |  |  |  |  |  |
| AQ25 |  |  | 0.127 |  | 0.103 |  |  | 0.1 |  | 0.343 |  |
| AQ26* |  | -0.162 |  | -0.151 |  |  | -0.152 | -0.151 | -0.152 |  |  |
| AQ27 |  |  |  |  |  |  |  |  |  |  |  |
| AQ28 | 0.365 |  |  |  |  | 0.24 | 0.357 | 0.245 |  |  |  |
| AQ29 | 0.126 |  |  |  |  |  | 0.094 |  |  |  |  |
| AQ30 | 0.237 |  |  |  |  |  |  |  | 0.258 |  |  |
| AQ31 |  |  |  |  |  |  |  |  |  |  |  |
| AQ32 | -0.541 |  | -0.429 |  |  | -0.327 | -0.455 | -0.321 | -0.312 | -0.606 |  |
| AQ33* | 0.113 |  |  |  |  |  | 0.109 |  | 0.117 |  |  |
| AQ34 | -0.12 |  |  | 0.053 |  |  |  |  |  | 0.191 |  |
| AQ35* | 0.562 | 0.443 |  | 0.55 |  |  | 0.559 |  |  |  |  |
| AQ36 | -0.171 |  | -0.176 | -0.076 | -0.072 | -0.075 | -0.063 | -0.068 | -0.055 | -0.101 |  |
| AQ37 | -0.073 |  | 0.057 |  |  | 0.168 |  | 0.174 |  | 0.228 |  |
| AQ38 |  | -0.174 |  | -0.159 | -0.158 |  | -0.159 | -0.158 | -0.159 |  |  |
| AQ39* | 0.122 |  |  | 0.109 |  |  |  | 0.128 |  |  |  |
| AQ40 | -0.064 | -0.193 |  |  |  |  |  |  |  |  |  |
| AQ41* | -0.198 |  | -0.202 | -0.12 |  | -0.258 | -0.205 | -0.283 | -0.132 | -0.321 |  |
| AQ42* | 0.049 |  | -0.101 |  |  |  |  |  |  |  |  |
| AQ43* |  |  |  |  |  |  |  | 0.06 |  |  |  |
| AQ44 |  |  |  |  |  |  |  |  |  | 0.263 |  |
| AQ45* |  |  |  | 0.122 | 0.126 | 0.123 | 0.136 | 0.131 | 0.144 | 0.176 |  |
| AQ46* |  |  | 0.154 | 0.199 |  |  | 0.197 | 0.197 | 0.199 | 0.418 |  |
| AQ47 |  |  |  |  |  |  |  |  |  | 0.24 |  |
| AQ48 |  |  |  | 0.066 |  |  |  |  | 0.082 |  |  |
| AQ49 | 0.403 |  |  |  |  |  |  |  |  |  |  |
| AQ50 |  | -0.114 | -0.12 |  |  |  |  | -0.106 | -0.161 |  |  |

All direct effects are significant; ^*^ Item is reverse coded.
